# Rational Design of Multifunctional Hydrogels for Wound Repair

**DOI:** 10.3390/jfb14110553

**Published:** 2023-11-18

**Authors:** Juan Cao, Bo Wu, Ping Yuan, Yeqi Liu, Cheng Hu

**Affiliations:** 1 School of Fashion and Design Art, Sichuan Normal University, Chengdu 610066, China; j.cao@sicnu.edu.cn; 2 School of Mechanical Engineering, Sichuan University, Chengdu 610065, China; scu_wubo@stu.scu.edu.cn (B.W.); 2022323020011@stu.scu.edu.cn (Y.L.); 3 School of Mechanical Engineering, Chengdu University, Chengdu 610106, China; yuanping@cdu.edu.cn; 4 National Engineering Research Center for Biomaterials, Sichuan University, Chengdu 610065, China

**Keywords:** multifunctional, hydrogel, microenvironment, wound repair

## Abstract

The intricate microenvironment at the wound site, coupled with the multi-phase nature of the healing process, pose significant challenges to the development of wound repair treatments. In recent years, applying the distinctive benefits of hydrogels to the development of wound repair strategies has yielded some promising results. Multifunctional hydrogels, by meeting the different requirements of wound healing stages, have greatly improved the healing effectiveness of chronic wounds, offering immense potential in wound repair applications. This review summarized the recent research and applications of multifunctional hydrogels in wound repair. The focus was placed on the research progress of diverse multifunctional hydrogels, and their mechanisms of action at different stages of wound repair were discussed in detail. Through a comprehensive analysis, we found that multifunctional hydrogels play an indispensable role in the process of wound repair by providing a moist environment, controlling inflammation, promoting angiogenesis, and effectively preventing infection. However, further implementation of multifunctional hydrogel-based therapeutic strategies also faces various challenges, such as the contradiction between the complexity of multifunctionality and the simplicity required for clinical translation and application. In the future, we should work to address these challenges, further optimize the design and preparation of multifunctional hydrogels, enhance their effectiveness in wound repair, and promote their widespread application in clinical practice.

## 1. Introduction

The skin, a crucial organ that envelops the body’s surface and directly interacts with the external surroundings, serves multiple functions, including sensing external stimuli, maintaining body temperature, and safeguarding the body against external harm. Damage to its structural integrity and function can lead to diverse forms of wounds. Research indicated that the process of skin wound repair is recognized as a dynamic and intricate journey, encompassing four consecutive stages, primarily categorized as hemostasis, inflammation, proliferation, and tissue remodeling [1,2,3,4]. In the early stage of skin damage, platelets are recruited to the trauma site and begin to aggregate while releasing thrombin to initiate the coagulation cascade reaction, catalyzing the conversion of fibrinogen to fibrin, which combines with aggregated platelets to form a blood clot. During the inflammatory phase, the release of inflammatory factors triggers the recruitment of neutrophils and macrophages to the injury site to phagocytose necrotic tissue, foreign debris and bacteria, and to provide local hemostasis. The proliferative phase is primarily characterized by the development of granulation tissue, neovascularization, the deposition of the extracellular matrix (ECM), and the re-epithelialization of the neo-epidermis. In the proliferative phase, newly generated blood vessels are embedded into the granulation tissue, providing ample oxygen and nutrients for cellular activity. Cytokines further activate fibroblasts, thereby enhancing cell proliferation and stimulating the secretion of collagen into the ECM. Epithelial cells begin migrating inward from the wound edge, ultimately covering the entire wound area. Finally, during the wound remodeling process, various cellular components (primarily macrophages), growth factors, as well as the nerve and immune systems, work in concert to regulate the synthesis and degradation of connective tissue. This allows for the repetitive dissolution, deposition, and renewal of collagen, leading to the gradual disappearance of scars and the continuous restructuring of the ECM, ultimately resulting in complete wound healing [5,6,7]. Currently, skin wound repair continues to be a prominent and formidable subject in both clinical and scientific research.

Common wound treatment strategies include ultrasound [8], electrotherapy [9], hyperbaric oxygen therapy [10], negative pressure therapy [11], stem cell therapy [12], dressings containing growth factors, etc. For skin injuries that involve the deeper layers of the dermis but are not extensive in size, if tissue repair can be accelerated and scar formation reduced using skin dressings, it would greatly facilitate the clinical treatment of skin injuries. Gauze, as a traditional dry dressing, is still widely used in clinical practice. However, it tends to adhere to the wound site, cause discomfort when removed due to its high water absorption, and offer limited protection against microbial invasion [13]. An ideal skin wound dressing should possess good tissue compatibility and moisturizing properties and be able to absorb tissue exudate while also having a certain level of mechanical strength, tissue adhesiveness, and surface microstructure [14,15]. It should stably remain on the wound surface, preventing external contamination, inhibiting bacterial growth, and promoting cell adhesion, proliferation, and differentiation [16,17]. Based on the aforementioned characteristics, hydrogels have gained prominence as the most promising skin wound dressings among numerous candidate materials due to their soft consistency, high porosity, excellent biocompatibility, and resilience [18,19,20]. It can maintain a moist environment for the wound, promoting cell regeneration and wound healing. Additionally, the excellent adhesive property of the hydrogel allows it to tightly adhere to the wound surface without causing secondary damage due to easy detachment. Most importantly, the hydrogel dressing possesses unique permeation regulation capabilities, enabling it to automatically adjust the permeation rate based on the wound condition, providing appropriate humidity and oxygen supply, thus accelerating wound healing. With innovative advancements in hydrogel design and synthesis techniques, coupled with in-depth research into skin wound repair mechanisms, the functionality of hydrogels has evolved from their early role of simple wound coverage to today’s multifunctional and intelligent repair capabilities, and the types of hydrogel dressings have also shown a trend of increasing year by year.

**Figure 1:**
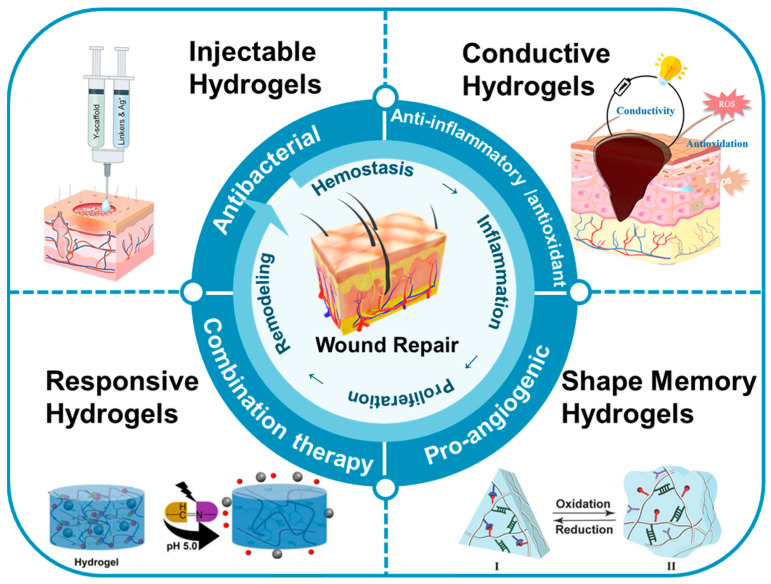
The applications of multifunctional hydrogels in the context of wound repair.

This review focused on the recent research and advancements in utilizing multifunctional hydrogels for wound repair (Figure 1). Firstly, we summarize the preparation and mechanisms of various types of multifunctional hydrogels, including injectable, responsive, conductive, shape-memory, and other types of hydrogels. Secondly, we discuss the diverse roles of these multifunctional hydrogels in wound repair, encompassing antibacterial, anti-inflammatory, antioxidant, pro-angiogenic, and combined therapeutic effects, demonstrating their efficacy. Finally, considering the current research landscape and clinical needs, we address the challenges associated with the use of multifunctional hydrogels in wound repair, with the objective of offering valuable insights for future research and applications of multifunctional hydrogels in the field.

## 2. Types of Multifunctional Hydrogels

Hydrogels are large polymer molecules composed of a network of crosslinked polymer chains [21]. The physicochemical properties and network structures of hydrogels vary depending on the preparation methods employed [22]. Based on the different crosslinking mechanisms, the preparation methods of hydrogels can be broadly classified into two categories: physical crosslinking and chemical crosslinking [23]. Physical crosslinking mainly involves non-covalent bonding interactions such as hydrophobic interactions [24,25], hydrogen bonding [26], subject–object interactions [27], electrostatic interactions [28], and biomolecular recognition [29]. These interactions are relatively mild and fast in response, but they are typically reversible, capable of being disrupted and restored under certain conditions, thus imparting shear-thinning and self-healing properties to the gel. However, hydrogels that are purely physically crosslinked tend to have lower mechanical strength and poorer stability. Chemical crosslinking refers to the formation of a hydrogel network through the establishment of covalent bonds. Common chemical crosslinking strategies include free radical polymerization [30], click reactions [31], Schiff base formation as dynamic covalent bonds [32], and enzymatic crosslinking reactions [33]. In comparison to physical crosslinking, chemical crosslinking typically requires precise structural design of the polymers, and the gelation rate and mechanical properties are directly influenced by the chemical reactivity between specific functional groups. Chemical crosslinked hydrogels exhibit higher strength and stability. However, they can also result in residual initiators, crosslinking agents, and unreacted monomers, which can potentially induce adverse reactions in living organisms and reduce the biocompatibility of the synthesized hydrogel materials. Therefore, they are not suitable for medical applications that require high biocompatibility standards.

The preparation method of hydrogels is a crucial factor that determines their physicochemical properties and applications. Researchers have developed various advanced functional hydrogels by applying different preparation methods to meet the different requirements of wound healing at different stages. With the pursuit of precision therapy, the development of more advanced hydrogels has attracted considerable attention. Different types of skin wounds have different shapes (size, thickness, etc.) and clinical manifestations (necrosis, decay, etc.), and the ideal hydrogel wound dressing should have the functions of rapidly forming an anti-infective barrier, promoting rapid blood coagulation, absorbing wound exudate, blocking nerve endings to reduce pain, and providing nutrients to promote tissue regeneration, etc. To meet these varying requirements for wound repair, researchers have developed various functional types of hydrogels, and the common ones include injectable, smart-response, conductive, and shape-memory.

### 2.1. Injectable Hydrogels

**Figure 2:**
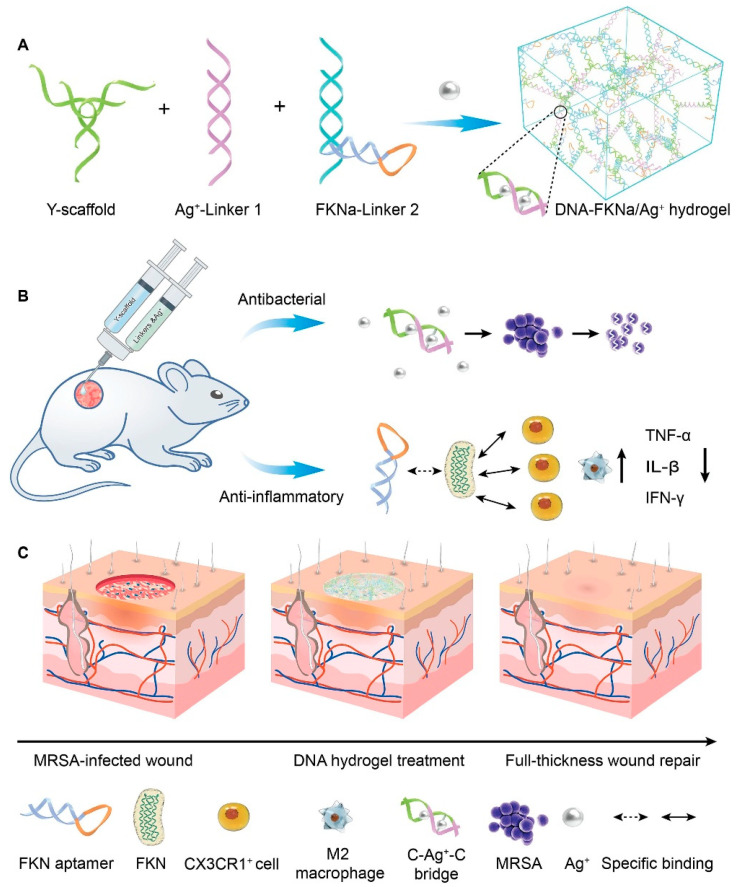
The schematic diagram depicts the DNA-FKNa/Ag^+^ hydrogel, which is designed for the treatment of *methicillin-resistant staphylococcus aureus* (*MRSA*)-infected wounds. (**A**) The compositions of the DNA-FKNa/Ag^+^ hydrogel. The enlarged picture presents the structure of C-Ag^+^-C bridges. (**B**) DNA-FKNa/Ag^+^ hydrogels were formed in situ by injecting DNA subunits into the bacterial infection defect using a three-way syringe. (**C**) *MRSA*-infected full-thickness wound repair using the dual functionalized DNA-FKNa/Ag^+^ hydrogel in an animal experiment. Adapted with permission from Ref. [40]. Copyright 2023 Elsevier.

The significant advantage of injectable hydrogels is their ability to conform to irregular wound shapes and serve as a platform for the delivery of drugs/cells and bioactive molecules (genes, proteins, growth factors, etc.), rendering them exceptionally promising for various wound repair applications. Injectable hydrogels can be categorized into two distinct types based on their gelation state and injection behavior: in situ gel-forming and shear-thinning hydrogels [34,35]. Among them, in situ gel-forming hydrogels mainly form a polymer network through certain physical effects or chemical crosslinking of two or more precursor solutions in vivo. A shear-thinning hydrogel is in a solid state (formed through reversible physical crosslinking) before injection. It transforms into a sol under the action of shear force, allowing flow injection in the needle tube. After the injection is completed, it can quickly self-repair to its original gel state. Amphiphilic block copolymers have been widely reported for preparing injectable hydrogels through self-assembly behaviors [36]. For example, Segura et al. [37] synthesized a tetra-armed poly(ethylene glycol)-poly(propylene sulfide) (PEG-PPS) block copolymer, where the hydrophobic self-assembly of PPS at the ends of the molecular chains could act as physical crosslinks to form injectable hydrogels, and this dynamic physical action could confer the gel with both shear-thinning and injectable properties. Injectable hydrogels have been extensively investigated as viable cell delivery systems due to their capability to mimic the extracellular matrix, uniformly encapsulate cells, facilitate effective mass transfer, accommodate chemical and physical modifications, and enable minimally invasive delivery. For instance, Chen et al. [28] synthesized polyglutamic acid-polyethylene glycol-polyglutamic acid (PGA-PEG-PGA) and polylysine-polyethylene glycol-polylysine (PLL- PEG-PLL) two block polymers. Among them, polyglutamic acid and polylysine have negative and positive charges, respectively. After mixing the two, they can quickly form an injectable hydrogel by relying on electrostatic interaction to achieve cell loading and passed function. Hu et al. [38] synthesized an injectable hydrogel that can demonstrate controlled delivery of the drug curcumin (Cur) and customized recombinant humanized collagen type III, precisely targeting the site of myocardial infarction. Chi et al. [39] developed an injectable hydrogel using thioglutamic acid (γ-glutamic acid) (γ-PGA- SH) and oxidized glycidyl methacrylate modified hyaluronic acid (OHA-GMA). This hydrogel exhibited remarkable properties such as biodegradability, biocompatibility, self-healing ability, and robust mechanical strength. Additionally, it demonstrated the capability to regulate fibroblast migration and infiltration through stiffness modulation, serving as an effective in situ scaffold for skin tissue regeneration. Wang et al. [40] constructed a straightforward, injectable, and multifunctional hydrogel (DNA-FKNa/Ag^+^) dressing by grafting DNA subunits, comprising cytosine (C)-rich strands and an fractalkine aptamer (FKNa), and it demonstrates remarkable suitability and significant potential for clinical translation in promoting and accelerating the healing of a *methicillin-resistant staphylococcus aureus* (*MRSA*)-infected wound (Figure 2).

### 2.2. Responsive Hydrogels

**Figure 3:**
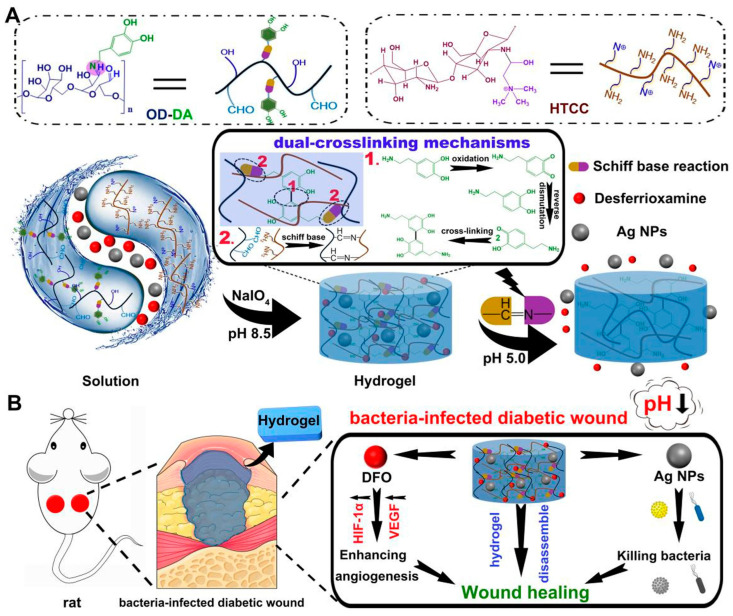
(**A**) The formation and mechanisms of the smart hydrogels. (1) Oxidative self-cross-linking mechanism of dopamine. (2) Schiff base cross-linking mechanism. (**B**) The antibacterial and wound healing mechanisms of the smart hydrogels. Reprinted with permission from Ref. [57]. Copyright 2023 Elsevier.

Responsive hydrogels are novel smart biomaterials capable of undergoing polymer chain conformational transitions or network changes under external environmental stimuli (e.g., specific temperature, light, pH, magnetic field, etc.), resulting in a series of specific responses, such as significant volume contraction/expansion, color change, and phase transition, making them injectable and self-healing with other properties [41]. These characteristics endow them with injectability, self-healing properties, shape memory, and more. The factors influencing wound healing include environmental factors (light, temperature, pH, CO_2_, etc.) that encounter the wound’s surface and physiological factors (cells, extracellular matrix, growth factors, ROS, enzymes, etc.) within the wound. Based on this, researchers have developed a series of responsive hydrogels in recent years, including physically responsive hydrogels responding to physical signals (temperature, light, electric fields, ultrasound, etc.) [42,43,44,45,46], chemically responsive hydrogels reacting to chemical signals (pH and ROS, etc.) [47,48,49,50], biologically responsive hydrogels responsive to biomolecules (enzymes and glucose, etc.) [51,52,53], and multi-responsive hydrogels [54,55]. For instance, Hu et al. [56] utilized caffeic acid-grafted ε-polylysine and phenylboronic acid-grafted oxidized dextran as the foundation to fabricate an injectable hydrogel. This hydrogel exhibits dual responsiveness to pH and ROS, and it encapsulates pH-responsive micelles (MIC@MF) that effectively promotes angiogenesis and anti-inflammatory DS. This feature facilitated the precise release of drugs at specific times and locations, aligning with the well-coordinated progression of wound healing in diabetic wounds. In another work, they exploited a pH-responsive, mussel-inspired, double-crosslinking injectable, and adhesive smart hydrogel to address the challenges associated with chronic diabetic wound repair. Importantly, the hydrogel exhibits antibacterial and angiogenesis-promoting characteristics, achieved through effective encapsulation of silver nanoparticles (AgNPs) and the pro-angiogenic drug deferoxamine (DFO), respectively [57] (Figure 3). Guo et al. [58] prepared a series of multi-stimulus response (NIR, ROS, and temperature) cryogels with controllable NO release capability based on methacryloyl carboxymethyl chitosan, poly(N-isopropylacrylamide), and composite nanoparticle enzyme (MSPA) for the adaptive treatment of drug-resistant bacterial-infected wounds.

### 2.3. Conductive Hydrogels

**Figure 4:**
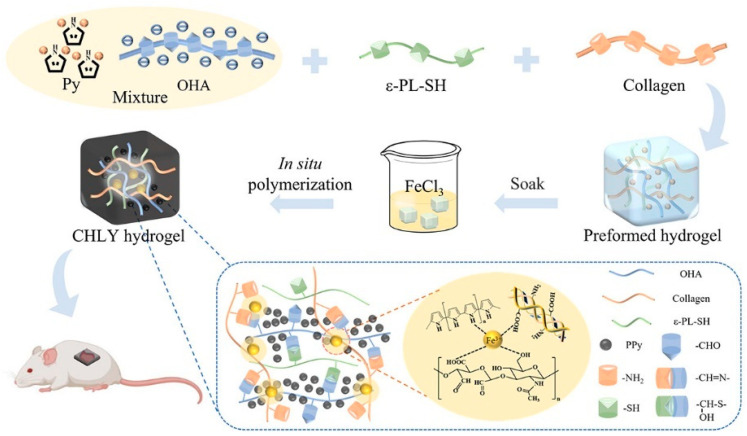
The preparation route of collagen-based hydrogels with multiple functionalities for inducing full-thickness wound healing. Adapted from Ref. [73].

Electrical stimulation expedites wound healing processes across all stages by engaging in multiple ways [59,60]. It relieves peri-electrode edema, directs keratinocyte migration, enhances re-epithelialization, directs cutaneous angiogenesis, modulates a range of genes related to wound healing, and produces antimicrobial action [61,62]. Consequently, strategies related to electrical stimulation for wound treatment have emerged. Conductive hydrogel is a novel composite material that combines a hydrophilic matrix organically combined with conductive fillers. Hydrogel with appropriate conductivity can maintain the wet environment of skin-defective wounds in a variety of mammals and enhance the electrical signaling of wounds, re-establish physiological functions related to electrical conduction, and promote wound healing [6]. The conductive mechanism can be classified into two categories: The first is through the introduction of ionic conductive materials (salt solution, ionic liquids, or polyelectrolytes) into the hydrogel network, in which the t network structure of the hydrogel provides a channel for the migration of ions so that the free ions are transmitted within it, and ultimately achieve ionic conductivity [63,64]. Secondly, it is a combination of electronically conductive polymers (ECPs) [65,66], carbon-based materials (e.g., carbon nanotubes and graphene oxide (GO)) [67,68], MXene [69], and metal-based materials [70], to establish the network of electron transport in hydrogel and realize electronically conductive. As an instance, Zhao et al. [71] presented a self-healing and highly conductive organogel composite. This composite structure was constructed by incorporating a permeable network of Ag microflakes and Ga liquid metal (LM) alloy microdroplets into a poly (vinyl alcohol)-sodium borate gel. It demonstrated remarkable conductivity of 7 × 10^4^ S m^−1^ and exhibited rapid and effective self-healing properties. Guo et al. [72] proposed a “deswelling in situ aggregation” method to induce colloidal particles of conductive polymer (PEDOT: PSS) to in situ aggregate into a continuous conductive network within a polyvinyl alcohol (PVA) network, resulting in a hydrogel with high electrical conductivity and high stretchability (breaking strain of 150%). Recently, Ge et al. [73] successfully created collagen-based hydrogels (CHLY) with multiple functionalities for inducing full-thickness wound healing. They achieved this by incorporating cysteine-modified ε-poly(l-lysine) (ε-PL-SH) and in situ-polymerized polypyrrole (PPy) nanoparticles into the hydrogel formulation (Figure 4). The resulting hydrogels demonstrated adhesive properties, conductivity, as well as antibacterial and antioxidant activities, making them highly versatile in promoting wound healing. Zuo et al. [74] successfully constructed SF/TA@PPy conductive hydrogels with stretchability, skin compliance, antimicrobial properties, and biocompatibility by introducing the conductive polymer polypyrrole (PPy) into the same gel network with filipin protein (SF) and tannic acid (TA) via in situ polymerization. Guo et al. [75] constructed a series of double dynamic bonded crosslinked hydrogels by combining sodium alginate oxide with dopamine/carboxymethyl chitosan/Fe^3+^. These hydrogels exhibited excellent electrical conductivity, self-healing capabilities, and photothermal antibacterial properties, thereby significantly enhancing the process of wound healing.

### 2.4. Shape Memory Hydrogels

**Figure 5:**
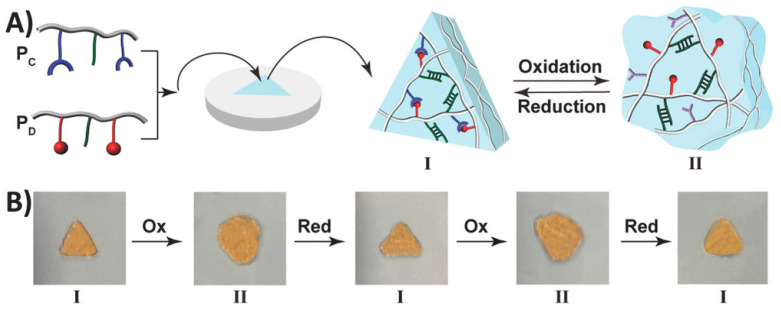
(**A**) The formation of a triangle-shaped hydrogel. (**B**) The redox-switchable transitions and shape-memory functionality of the hydrogel. Reprinted with permission from Ref. [84]. Copyright 2023 John Wiley and Sons.

Shape memory hydrogels (SMHs) can retain a temporary shape and restore their initial shape under specific stimuli, displaying a shape memory capability [76,77]. They have extensive applications in various fields, including drug delivery, 3D printing, tissue engineering, and sensors [78,79]. SMHs rely on two special crosslinking structures within their 3D network to achieve shape memory functionality: The first type of crosslinking structure is called permanent crosslinking (e.g., irreversible chemical bonds), and SMHs form their initial shape under the action of this crosslinking agent. The second crosslinking structure is reversible dynamic crosslinking characterized by the stimulus response [76,80,81], including hydrogen bonds, host–guest interactions, coordination interactions, and reversible chemical bonds (dynamic borate bonds, dynamic Schiff base bonds, etc.). This dynamic crosslinking structure allows SMHs to break and reassemble reversibly under external forces, giving them the ability of shape memory. For example, Liu et al. [82] constructed a novel type of radiopaque dual-stimulus-responsive shape memory hydrogels through a straightforward one-step polymerization process. The hydrogels were prepared by combining acrylonitrile (AN), N-acryloyl 2-glycine (ACG), and the crosslinker poly(ethylene glycol) diacrylate (Mn = 700, PEGDA_700_). Mano et al. [83] reported a facile method to convert non-thermally responsive hydrogels into thermally responsive hydrogel systems with shape memory capability. As a proof of concept, they provided hydrogel composites with shape memory capabilities by embedding polyurethane networks in heat-sensitive polyurethane chitosan methacrylate, gelatin, laminin, or hyaluronic acid hydrogel. Along this line, researchers have developed shape memory hydrogels with a high shape fixation rate (50–90%) and exceptional shape recovery rate (nearly 100% with almost instantaneous recovery). Willner et al. [84] presented a redox switchable shape memory hydrogel system composed of bipyridinium, which complexes as crosslinking units of carboxymethyl cellulose and dopamine (Figure 5). Xing et al. [85] reported the development of a physical crosslinked PVA hydrogel by introducing a high concentration of sodium hydroxide to a high-density PVA polymer, thereby inducing crystallization. The resulting hydrogel exhibited remarkable mechanical properties, reduced water content, enhanced damage resistance, and demonstrated shape memory capabilities.

## 3. Applications in Wound Repair

Combining the sequential stages of wound healing and their distinctive characteristics, hydrogel-based wound repair strategies often focus on antimicrobial, anti-inflammatory, antioxidant, pro-vascularization, and a combination of multiple therapeutic approaches to promote rapid wound healing, as shown in Table 1.

### 3.1. Antibacterial

**Figure 6:**
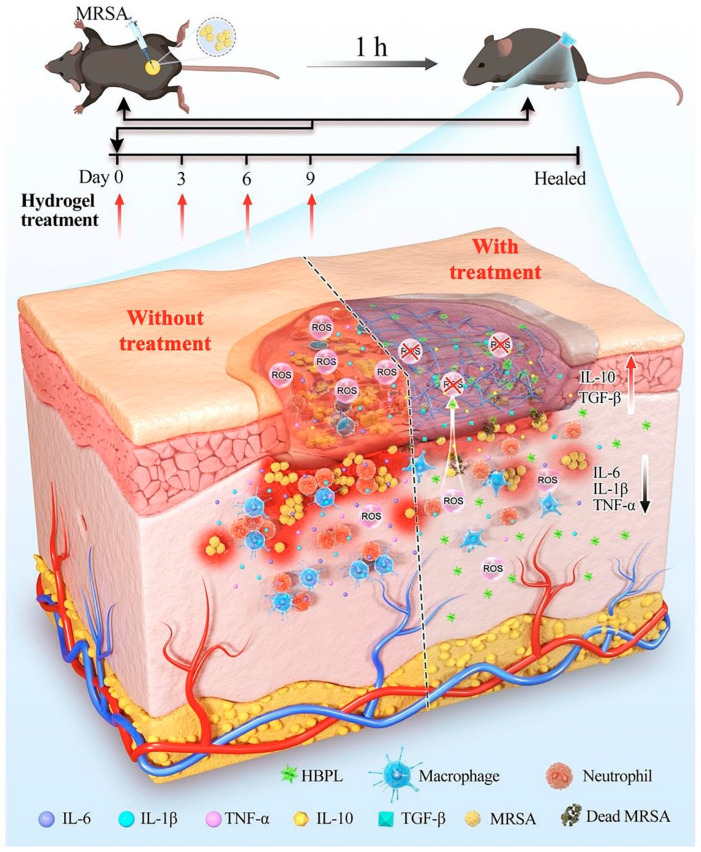
The mechanism of the ROS-scavenging hydrogel containing hyperbranched poly-L-lysine (HBPL) promoting wound healing. Reprinted with permission from Ref. [86]. Copyright 2023 Elsevier.

The occurrence of bacterial infections during wound healing presents an unavoidable and urgent challenge. Regrettably, the improper utilization of antibiotics has resulted in the emergence of multidrug-resistant bacteria, thereby exacerbating the already formidable challenges associated with antimicrobial therapy for wound treatment. Addressing the medical bottleneck of achieving efficient antimicrobial efficacy while effectively promoting the wound healing process remains a paramount challenge that necessitates the collective efforts of researchers. There are many kinds of antimicrobial materials, including antibiotics, metal ions (Ag^+^, Cu^2+^, etc.), cationic polymers (quaternized chitosan), biomimetic nano-enzymes (MoS_2_), antimicrobial peptides, etc. [99,100]. Antimicrobial methods also include chemodynamic therapy (CDT), phototherapy (PDT, PTT), and magnetic hyperthermia therapy (MHT), etc. [101,102]. Researchers have constructed corresponding antimicrobial hydrogels to effectively promote healing [22,103]. *S. aureus* stands as the primary pathogen responsible for skin infections, and the presence and dissemination of *MRSA* have presented a significant hurdle in treating wound infections due to its formidable drug resistance and potent virulence. Moreover, the excessive production of reactive ROS at the site of skin wounds exacerbates inflammation, resulting in delayed healing and extensive scarring. Drawing from this information, Lu et al. [86] designed an ROS-scavenging hydrogel containing hyperbranched poly-L-lysine (HBPL), a bacterial population-sensing inhibitor, demonstrating effective elimination of *MRSA*, whether in planktonic form or within biofilm structures. In vivo, this hydrogel effectively promoted the healing of *MRSA*-infected whole skin defects by impeding quorum sensing (QS), eradicating bacteria, and suppressing inflammation, ultimately promoting the healing process, and this study provided new insights into scarless healing of methicillin-resistant *S. aureus*-infected skin wounds (Figure 6). Hu et al. [56,104,105,106,107,108] developed a series of smart-responsive hydrogels loaded with antimicrobial drugs for on-demand, controlled release of drugs at the site of the wound to facilitate the healing process. Guo et al. [87] reported a self-healing hydrogel that possessed favorable electrical conductivity and antimicrobial properties. The hydrogel was fabricated using quaternized chitosan (QCS), oxidized dextran (OD), tobramycin (TOB), and surface-modified polypyrrole nanowires with polydopamine (PPY@PDA NWs). Additionally, it was crosslinked by Schiff base and could thus achieve the on-demand release of TOB in response to weak acidic pH, killing high concentrations of *PA* and *S. aureus* within a short period of time and promoting wound healing. Li et al. [88] integrated iron oxide nanoparticles loaded with polydopamine (PDA) with glucose oxidase (GO_x_) and hyaluronic acid (HA) grafting onto microneedle (MN) patches and introduced amine-modified mesoporous silica nanoparticles (AP-MSN) into the substrate, resulting in PFG/M microneedle patches. The experimental results showed that the microneedle patch combined chemical dynamic therapy (CDT), photothermal therapy (PTT), and tip-Fe/PDA@GOx@HA-induced M2 macrophage polarization, exhibiting excellent antimicrobial and immunomodulatory properties. This innovative development holds great promise as a potential clinical candidate for the treatment of infected wounds.

### 3.2. Anti-Inflammatory and Antioxidant

**Figure 7:**
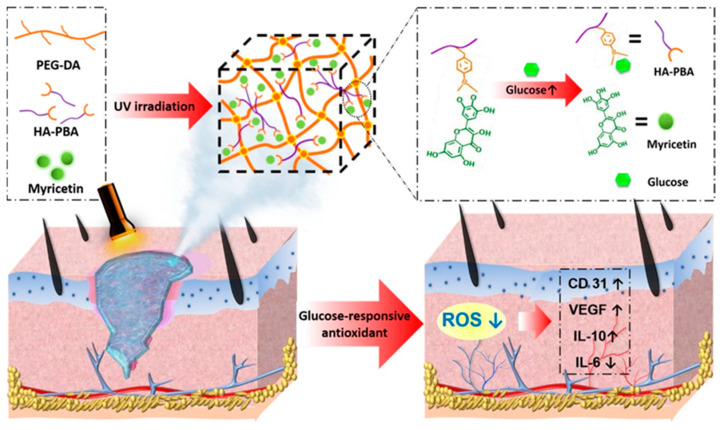
The preparation process of PHM hydrogel and its mechanism of diabetic wound repair. Reprinted with permission from Ref. [90]. Copyright 2023 American Chemical Society.

During the second stage of wound healing, known as inflammation, immune cells secrete various inflammatory mediators such as cytokines, chemokines, adhesion molecules, etc., to combat bacterial or viral infections and eliminate factors causing physical damage. However, at the same time, oxygen consumption increases, and the accumulation of reactive ROS generated by the “respiratory burst” can lead to DNA damage and apoptosis, further exacerbating inflammation and affecting wound healing. Appropriate inflammation is essential in wound repair, and therefore the regulation of inflammation is important for wound repair. Antioxidants can aid in capturing and neutralizing free radicals, thus eliminating their damaging substances to the organism [109]. The incorporation of well-known natural antioxidants, such as catechins [110], resveratrol [111], anthocyanins [112], and some flavonoids [113], etc., into hydrogels has been employed to harness their antioxidant properties and facilitate the process of wound healing. For example, Xiao et al. [89] developed an injectable self-repairing hydrogel with inherent antimicrobial properties by utilizing the dynamic covalent bond formation between boric acid and catechol moieties within quaternized chitosan as a masonry block, coupled with the in situ encapsulation of epigallocatechin-3-gallate (EGCG). The authors evaluated the antioxidant efficiency of the hydrogel by measuring its ability to scavenge 1, 1-diphenyl-2-picrylhydrazyl (DPPH) free radicals. And the results revealed that the hydrogel demonstrated remarkable antioxidant effects. Wu et al. [90] developed an innovative composite hydrogel with glucose-responsive and antioxidant properties, specifically designed for the purpose of diabetic wound repair. They first prepared glucose-sensitive phenylboronic acid (PBA)-modified hyaluronic acid (HA), which was then combined with polyethylene glycol diacrylate (PEG-DA) to form a novel composite hydrogel (PEG-DA/HAPBA). Next, the researchers immobilized poplar plum flavonoid (MY) molecules, known for their potent antioxidant activity, within the hybrid hydrogel (Figure 7). By measuring the DPPH clearance rate and the ROS indicator 2′,7′-dichlorodihydrofluorescein diacetate (DCFH-DA), the PEG-DA/HA-PBA/MY (PHM) hydrogel was found to effectively scavenge ROS (>80.0%) and revitalize the microenvironment of oxidative wounds. Over the past few years, a series of nanomaterials that can mimic natural antioxidants have been created, such as cerium oxide nanoparticles, iron oxide nanoparticles, and carbon nanomaterials (ceria (CeO_2_) nanoparticles and manganese oxide (MnO_2_, Mn_2_O_3_, Mn_3_O_4_, and MnO_2_) nanoparticles and carbon nanomaterials), etc. These materials, compared to natural antioxidants, maintain high stability in more complex disease environments [114]. Incorporating them into hydrogels can effectively eliminate ROS at the wound site and promote wound healing [115,116,117]. For instance, Lei et al. [91] prepared multiple coordination-derived bioactive hydrogels (SGPA) by simple multi-metal coordination using sodium alginate, metal ions (Gd^3+^), and phosphoric acid-functionalized polycitric acid as the raw materials. SGPA has good injectability, self-healing properties, and controlled biodegradability. In addition, it exhibits favorable cytocompatibility and hemocompatibility while also enhancing the migration of endothelial cells. Moreover, the SGPA hydrogel demonstrated notable hemostatic efficacy in an in vivo liver hemorrhage model. In the complete skin wound model, the SGPA hydrogel demonstrated significant effectiveness in promoting wound healing by reducing the expression of inflammatory factors and stimulating angiogenesis in the peri-wound area. Xie et al. [92] developed an in situ hydrogel with a combination of antibacterial, antioxidant, immunomodulatory, and wound-adaptive characteristics specifically for the effective treatment of infectious wounds. The hydrogel formulation comprises two naturally derived biopolysaccharides, namely sodium alginate (SA) and carboxymethyl chitosan (CMCS). By employing “click” chemical reactions, Schiff base reactions, and other reaction principles, modified derivatives such as sodium maleimido oxidized alginate (AM) and mercapto carboxymethyl chitosan (CS) are crosslinked into gum A_x_C_y_ (x, y = 3, 4, 5, 6, 7; x + y = 10), which exhibits certain ROS scavenging abilities and can play a pro-infectious wound healing role through resistance to *E. coli*, *S. aureus* infection, antioxidant, and macrophage polarization modulation properties.

### 3.3. Pro-Angiogenic

**Figure 8:**
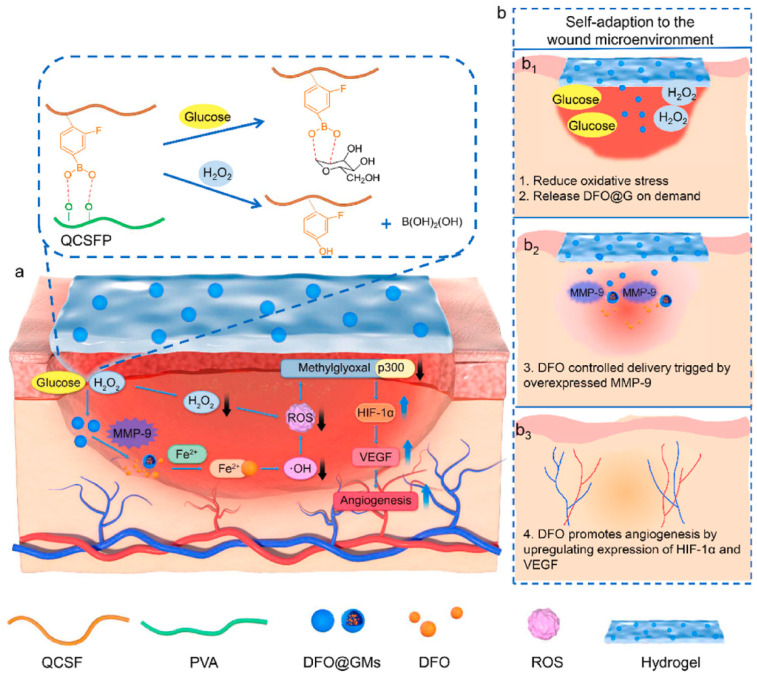
The preparation of the DFO@G-QCSFP hydrogel and its mechanism for accelerating diabetic wound healing. (**a**) The chemical structure of the hydrogel and the mechanism of the hydrogel for accelerating diabetic wound healing. (**b**) The self-adaption of the hydrogel to wound microenvironment. The hydrogel reduced oxidative stress and released DFO@G on demand. Then DFO was released and promoted angiogenesis. Reprinted from Ref. [93].

Blood vessels are involved in the transportation of oxygen and other nutrients required for wound healing; therefore, neovascularization is essential for the healing of wounds, and it can be argued that the timely development of blood vessels in the early stages is inherently linked to the rate of wound healing [118]. Over the years, there has been extensive research into the use of growth factors to induce angiogenesis and enhance wound closure in chronic wounds. However, treatment methods based on growth factors have limitations, such as high cost and a short half-life, which restrict their application [119]. Utilizing hydrogels as carriers to deliver small-molecule drugs, adipose-derived mesenchymal stem cells, and other substances that promote vascular formation has become a common treatment for diabetes-related wounds with poor neovascularization [120]. For instance, Xu et al. [121] prepared a hyperbranched poly (β-amino ester) hydrogel using the Schiff base reaction between an amine and acrylic ester. This polyethylene glycol-based hydrogel can undergo controlled degradation and be loaded with adipose-derived mesenchymal stem cells. The measurement of the number of blood vessels formed and the expression levels of VEGF in the wound after treatment with this hydrogel revealed that it effectively promoted angiogenesis and accelerated the healing of diabetic wounds. DFO is an iron chelator that has been approved by the FDA for clinical use. Previous studies have demonstrated that DFO can significantly accelerate the formation of new blood vessels under normal and pathological conditions by upregulating the expression of hypoxia-inducible factor-1α (HIF-1α) and its downstream gene VEGF [122,123]. Zhou et al. [93] synthesized quaternized chitosan (QCS) and then grafted 3-carboxy-4-fluorophenylboronic acid onto the QCS side chains, creating QCSF with grafted phenylboronic acid groups. They used the dynamic boronate ester bonds between the phenylboronic acid groups on QCSF and the hydroxyl groups on polyvinyl alcohol (PVA) to develop a drug delivery system involving crosslinked gelatin microspheres for the drug DFO. This DFO-loaded DFO@G-QCSFP hydrogel dynamically regulated the microenvironment by scavenging ROS and releasing DFO as needed, thus promoting vascular regeneration and diabetes wound healing (Figure 8). Guo et al. [94] constructed a pH/glucose dual-responsive metformin hydrogel dressing based on the dual dynamic bonds formed by Schiff base and boronic acid ester. The Schiff base structure possesses pH sensitivity and exhibits instability in acidic conditions, leading to enhanced drug release. The neighboring phenolic structure has the ability to form dynamic boronate ester structures with boronic acid, responding to glucose. The results of immunofluorescence staining demonstrated that treatment with the hydrogel significantly reduced the levels of the pro-inflammatory cytokine interleukin-6 (IL-6) in the wound and markedly increased the number of newly formed blood vessels. This dual-responsive hydrogel improved wound healing in a rat model of type 2 diabetes by inhibiting inflammation and promoting vascular regeneration. Nitric oxide (NO) has been identified as a crucial molecule in wound healing, playing a vital role in vascular regeneration. Guo et al. [124] developed a multifunctional conductive hydrogel with the ability to release NO under near-infrared laser irradiation to accelerate vascular formation and wound healing. Researchers have found that appropriate thermal stimulation can enhance granulation tissue growth, promote vascular formation, and expedite skin wound healing [125]. Wang et al. [126] developed an injectable, self-healing hydrogel formed through Schiff base bonds between poly (ε-L-lysine) and oxidized hyaluronic acid, leveraging the thermal responsiveness of Pluronic F127. By incorporating exosomes into this hydrogel, they effectively promoted neovascularization in chronic wounds, accelerating their healing. Ren et al. [95] prepared a hybrid hydrogel, Fe\PPHP15, with pro-angiogenic and targeted antibacterial properties. The phenol–iron complex (TA@Fe^3+^) in the hydrogel served as a photothermal conversion agent, converting light energy into gentle heat at an 808 nm excitation wavelength. The incorporation of iron ions in the hydrogel significantly facilitated the expression of genes related to blood vessel growth, such as bFGF, bFGFR, and HIF-1. The mild thermal effect additionally induced the expression of VEGF. The upregulation of these angiogenesis-related genes promoted vascular formation at the wound site.

### 3.4. Combination Therapy

**Figure 9:**
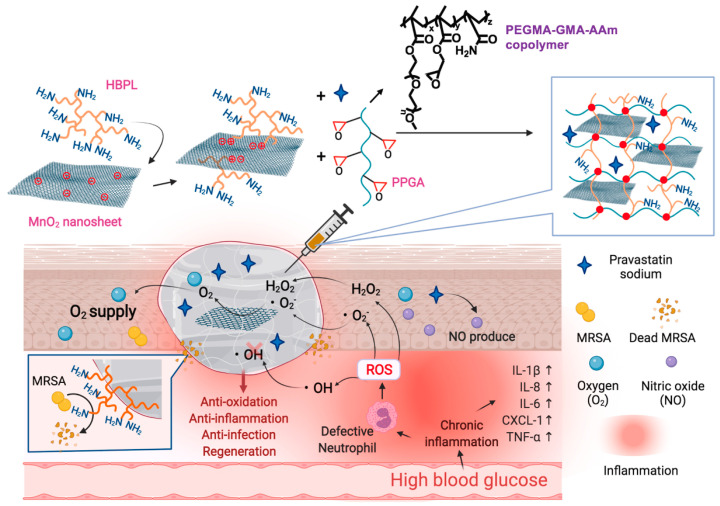
The chemical structure of the composite antibacterial and antioxidant hydrogel and its mechanism for accelerating diabetic wound healing. Reprinted with permission from Ref. [97]. Copyright 2023 Elsevier.

Wound healing is a complex process that can be impacted by diseases or infections in diverse ways, such as changes in inflammatory responses, altered ROS signaling, and more. Treating wound repair by addressing a single factor is insufficient. In recent years, researchers have developed a variety of combination therapies to address the challenges of wound healing. Wen et al. [127] designed a multifunctional DNA hydrogel by dynamically crosslinking non-immunogenic DNA with polyethyleneimine and incorporating heating-functionalized black phosphorus quantum dots. This DNA hydrogel exhibited remarkable adjustable heating capability, mechanical properties, self-healing ability, and antimicrobial properties. Furthermore, the incorporation of oligomeric proanthocyanidins B2 (OPC B2) endowed the DNA hydrogel with potent free radical scavenging and antioxidant properties. Additionally, the multifunctional DNA hydrogel dressing facilitated the transition of macrophages from the pro-inflammatory M1 phenotype to the repair-promoting M2 phenotype, thereby maintaining a stable remodeling state at the wound site. Moreover, the DNA hydrogel dressing activated neurons, inducing them to enter a reparative state and accelerating the regeneration of skin nerves and the formation of blood vessels. Chen et al. [96] prepared a hydrogel through coordination crosslinking of multi-arm thiolated polyethylene glycol with silver nitrate. This hydrogel’s self-healing and injectable properties stemmed from dynamic disulfide bonds and ionic coordination bonds. Silver ions endowed the hydrogel with antimicrobial properties. Furthermore, the authors successfully enhanced the healing of diabetic wounds by incorporating the pro-angiogenic drug DFO into the hydrogel. Gao et al. [97] developed a composite antibacterial and antioxidant hydrogel based on hyperbranched poly (β-amino ester) (HBPL) with inhibitory effects on bacterial quorum sensing and manganese dioxide nanosheets (Figure 9). They crosslinked this material with a poly (PEGMA-co-GMA-co-Aam) (PPGA) polymer to create a multifunctional injectable hydrogel dressing. This dressing was employed for effective antibacterial and antioxidant actions as well as continuous oxygen supply in the treatment of diabetic-infected wounds, achieving the goal of inflammation inhibition and repair promotion. Li et al. [98] designed a novel antibacterial and antioxidant dual-functional, low hysteresis, stretchable hydrogel (MPH) through copolymerization of N-isopropylacrylamide, acrylamide, and acrylate Pluronic 127 (PF127-DA), along with functionalization of molybdenum disulfide-polydopamine nanoparticles (MP). This hydrogel, suitable for close adhesion to moving body parts and wound healing, displayed excellent mechanical properties that allowed it to tightly adhere to the wound, resist the invasion of *E. coli* and *S. aureus*, and avoid secondary fixation of dressings. Furthermore, the hydrogel’s dual functionality, stemming from its photothermal characteristics and enzyme-like activity, effectively eliminated bacterial infections, mitigated oxidative stress, enhanced the wound microenvironment, and facilitated the process of wound healing.

## 4. Conclusions and Future Perspectives

The process of wound healing is intricate and time-consuming, significantly impacting the quality of life of countless individuals worldwide. Recently, significant progress has been made in the research of various multifunctional hydrogels, which also show great potential and application in wound repair. We reviewed the therapeutic strategies of multifunctional hydrogels in different stages of wound repair, such as antimicrobial, anti-inflammatory, antioxidant, pro-angiogenic, and a combination of multiple strategies, which provide strong support for wound healing. In addition, multifunctional hydrogels have been combined with other advanced fabrication technologies, such as 3D printing, for personalized wound repair strategies [128,129]. Designing flexible skin sensors is another direction that may significantly impact chronic wound therapy [130,131,132]. In this context, the utilization of hydrogel-based bioelectronic devices offers a promising platform for wound therapy. These devices have the capability to monitor the real-time status of wounds while also enabling the controlled and targeted release of bioactive molecules or drugs as needed.

However, we must also face the challenges faced by multifunctional hydrogels in practical applications, including how to rationally design and combine the functionality and therapeutic strategies of hydrogels, how to prepare hydrogels on a large scale and effectively preserve them, and how to design and screen them more efficiently with the help of artificial intelligence. In addition, patients are not only pursuing the filling of simple defects in the treatment of wounds, but they also have the demand for full functional recovery and aesthetics, and there remains ample opportunity for the further development of therapeutic strategies based on multifunctional hydrogels in the prevention and treatment of scarring, regeneration of hair follicles, and improvement of pigmentation abnormality. It is worth noting that achieving multifunctionality of hydrogels typically involves complex designs of hydrogels, which is contradictory to the simplicity required for clinical translational applications. Therefore, to further enhance the efficacy and clinical application of multifunctional hydrogels in wound repair, we need to actively explore advanced preparation techniques, expand wider application areas, and strengthen the integration of clinical practice and scientific research. Last but not least, multifunctional hydrogels used for wound repair must possess superior safety, biocompatibility, and non-immunogenicity for their clinical application. This ensures that the hydrogel, when in contact with human tissues, does not cause any harmful side effects, including allergies, tissue necrosis, inflammation, or excessive immune system reactions. Therefore, strict control over the selection of raw materials, preparation processes, and quality assurance is necessary to guarantee the effectiveness and safety of hydrogel in clinical use. Overall, the research and application of multifunctional hydrogels in wound repair will bring important innovations and advances to the medical field and substantially improve the health and quality of life of patients.

## Figures and Tables

**Table 1:** Hydrogels with different therapeutic strategies in wound repair.

Hydrogels	Functional Elements	Therapeutic Strategies			References
		Antibacterial (Bacterial Species)	Anti-Inflammatory and Antioxidant (Evaluating Test)	Pro-Angiogenic (Effect Cargos)	
PPG hydrogel	HBPL	+ *MRSA*			[86]
QCS/OD/TOB/PPY@PDA	TOB	+ *Pseudomonas aeruginosa (PA)*, *Staphylococcus aureus (S. aureus)*			[87]
PFG/M microneedle	polydopamine (PDA)-loaded iron oxide	+ *Escherichia coli (E. coli)*, *S. aureus*			[88]
quaternized chitosan hydrogel	quaternized chitosan and EGCG		+ 1,1-diphenyl-2-picrylhydrazyl (DPPH) free radical and reactive oxygen species assay		[89]
PEG-DA/HA-PBA hydrogel	myricetin		+ DPPH, 2′,7′-dichlorodihydrofluoresceindiacetate (DCFH-DA), interleukin-6 (IL-6), IL-10		[90]
SGPA hydrogel	poly(citrate-ethylene glycol-alendronate) (PCA) and Gd^3+^		+ c	+ PCA	[91]
maleimide-based oxidized sodium alginate and sulfhydryl carboxymethyl chitosan hydrogel	sodium alginate and sulfhydryl carboxymethyl chitosan	+ *E. coli*, *S. aureus*	+ DHE probe		[92]
DFO@G-QCSFP hydrogel	DFO			+ DFO	[93]
PEGS-PBA-BA/CS-DA-LAG hydrogel	metformin and graphene oxide		+ DPPH	+ metformin (Met)	[94]
Fe\PPHP15 hybrid hydrogel	Fe^2+^ and Fe^3+^	+ *E. coli*, *S. aureus*		+ Fe^2+^\Fe^3+^	[95]
Ag-SH-PEG hydrogel	Ag^+^ and DFO	+ *S. aureus*		+ DFO	[96]
HMP hydrogel	HBPL and NO	+ *MRSA*	+ DPPH, superoxide anion free radical (∙O_2_^−^), hydroxyl radical (∙OH)		[97]
MP composite hydrogel	molybdenum disulfide-polydopamine nanozyme	+ *E. coli*, *S. aureus*	+ ·OH scavenging efficiency, DCFH-DA		[98]
